# Investigation of the short-term in vivo performance of metal-on-carbon fibre reinforced poly ether ether ketone Motec wrists: an explant analysis

**DOI:** 10.1177/17531934241249919

**Published:** 2024-05-23

**Authors:** Thomas J. Joyce, Göksu Kandemir, David Warwick, Daniel J. Brown

**Affiliations:** 1 School of Engineering, Newcastle University, Newcastle upon Tyne, UK; 2 Orthopaedics, University Hospital, Southampton, UK; 3 Faculty of Health and Life Science, University of Liverpool, Liverpool, UK; 4 Liverpool University Hospitals NHS FT, Liverpool, UK

**Keywords:** Wrist replacement, wrist arthroplasty, wear, failure, indentation

## Abstract

Total wrist arthroplasty (TWA) aims to restore pain-free motion to diseased joints. One such TWA, the Motec, has demonstrated good results with acceptable complication rates. It has recently been suggested that the metal-on-carbon fibre reinforced poly ether ether ketone (Mo-CFR-PEEK) version of the Motec TWA be implanted instead of the metal-on-metal version. An explant analysis was undertaken on seven Motec Mo-CFR-PEEK TWAs, revised for a variety of reasons, after a mean time of 2 years in vivo. Compared to a new Motec implant, five of the explanted metal heads and three of the CFR-PEEK cups became smoother in vivo, suggesting self-polishing and negative skewness, indicating some material loss in vivo. Two explanted cups showed indentation marks on their rims and one of these was from component impingement with embedded metallic debris. In the short-term, the articulating surfaces of Motec Mo-CFR-PEEK TWAs did not show major damage.

**Level of evidence:** IV

## Introduction

Total wrist arthroplasty (TWA) has traditionally been associated with designs employing a metal-on-polymer (MoP) articulation in recipients diagnosed with rheumatoid arthritis (Berber et al., 2018). With the introduction of the Motec (Swemac Innovation AB, Linkoping, Sweden) TWA and its metal-on-metal (MoM) articulation, the aim was to offer TWA to younger, higher-demand recipients diagnosed with osteoarthritis. MoM was chosen as the preferred articulating surfaces at the time, as it was considered to be a more resilient construct than MoP.

The cementless Motec wrist is a fourth-generation wrist replacement that comprises a ball-and-socket articulation. The metal used for the MoM articulation is cobalt chromium (CoCr). This is a modular implant with the CoCr radial cup and the CoCr metacarpal head components fitting within Bonit® (resorbable calcium phosphate) coated titanium alloy ‘screws’ that provide both original fixation and subsequent osseointegration.

While positive results have been presented for the MoM Motec implant (Giwa et al., 2018; Reigstad et al., 2012, 2017), a recent paper suggested a move away from MoM. They recommended discontinuing the use of MoM in TWA and suggested instead using a MoP articulation, such as metal on poly ether ether ketone (PEEK) (Holm-Glad et al., 2022), although they accepted that there is no evidence to support this alternative. The current version from the manufacturer, Swemac (Swemac Innovation AB, Linkoping, Sweden) includes a version of the Motec TWA with a carbon-fibre reinforced poly ether ether ketone (CFR-PEEK) radial cup, paired with the usual CoCr metacarpal head giving a metal-on-CFR-PEEK (Mo-CFR-PEEK) articulation. Within orthopaedics, this is not a common bearing material combination.

A recent multicentre study looked in detail at 171 Motec TWAs at a mean follow-up of 5.8 years (Redfern et al., 2024). There were 113 MoM and 58 Mo-CFR-PEEK implants. No difference in complication rates between MoM and Mo-CFR-PEEK TWAs was found. Another way to examine the performance of different TWA articulations can be obtained through explant analysis, even when the implants have only been in vivo for a short time. The issue of impingement related osteolysis related to short-neck MoM Motec TWAs has been investigated and explant analysis has shown damage to the extra-articular components of the implant (Julian et al., 2024). A further explant analysis of the articulating surfaces of MoM Motec TWAs, none of which showed impingement related osteolysis, has recently been reported and demonstrated minimal articular damage and evidence of self-polishing (Kandemir et al., 2024). The aim of the present study was to investigate the performance of Mo-CFR-PEEK Motec TWAs through explant analysis of seven samples.

## Methods

All Motec Mo-CFR-PEEK wrist implants removed from patients, at the time of revision surgery, were cleaned and sterilized, using standard techniques in the on-site sterile services unit. The study was registered and approved by the departmental research committee and is in line with current Medical Device Regulation recommendations. Explants were then sent to a specialist university biomedical engineering department for analysis. One complete unused Mo-CFR-PEEK Motec wrist implant (all four components: metacarpal head, metacarpal screw, radial cup and radial screw) was provided by the manufacturer for comparison; this sample was termed ‘Motec 0’.

Data relating to patient demographics, diagnosis, time to revision, reason for revision, revision surgery performed and relevant radiographic images were obtained from electronic patient records (Bluespier, Droitwich, UK) and the picture archiving and communication system (Carestream Vue PACS, Rochester, NY, USA).

For each explant, the surfaces of the explanted heads and cups, and any available screws, were visually analysed and photographed to identify any surface damage. A Zygo NewView 5000 (Lambda Photometrics, Harpenden, UK) non-contacting profilometer (with a sensitivity of 0.001 µm, Sa) (Scholes et al., 2017) was used to measure the topographies of the articulating surfaces of the metacarpal heads and radial cups. Eight surface roughness (Sa) and skewness (Ssk) measurements were taken from each articulating surface. Due to the size of the Zygo lens, analysis was restricted to the central pole region of the radial cup.

All measurements taken from the articulating surfaces of the explants were compared to those from the unused Motec implant sample. The statistical relevance of the comparison was determined using a *t*-test with a 95% confidence interval and 0.05 significance.

## Results

Clinical details related to the explanted Mo-CFR-PEEK Motec TWAs are given in Table 1. The mean age of the seven patients at surgery was 61 years (range 52–70). Implantation was due to a variety of reasons including post-traumatic osteoarthritis (*n* = 3), primary osteoarthritis (*n* = 2) and Keinböck’s disease (*n* = 1).

**Table 1.** Clinical details of the explanted Mo-CFR-PEEK Motec TWAs.

Motec	Components available for analysis	Head neck size	Reason for implantation	Patient sex and age at surgery	Reason for removal	In vivo time (years)
1	H, C, MC and R screws	Long	OA	Male, 70	Failure of osseointegration	1.3
2	H, C, R screws	Medium	Post-traumatic OA	Female, 68	Failure of osseointegration	1.8
3	H, C, R screws	Long	OA	Male, 52	Incorrect centre of rotation	0.5
4	H, C, MC screws	Medium	Keinböck’s	Female, 53	Osteolysis following PEEK wear	3.8
5	H, C	Long	Post-traumatic OA	Male, 63	Ongoing pain	2.8
6	H, C	Long	Revision of MoM Motec	Female, 70	Osteolysis following PEEK wear	2.0
7	H, C, MC and R screws	Long	Post-traumatic OA	Male, 53	Failure of osseointegration	1.7

C: cup; H: head; MC: metacarpal; Mo-CFR-PEEK: metal-on-carbon fibre reinforced poly ether ether ketone; MoM: metal-on-metal; OA: osteoarthritis; R: radial; TWA: total wrist arthroplasty.

**Figure 1.**
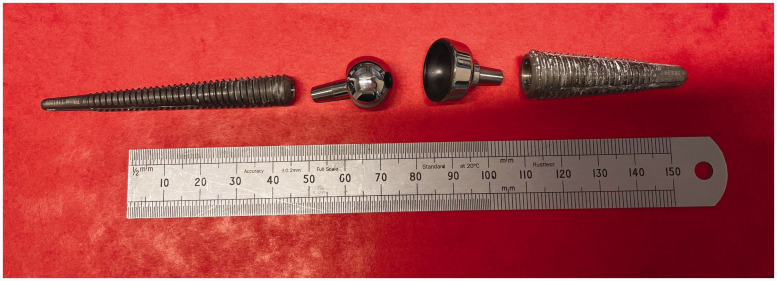
An image of the Motec 1 explant. From left to right, the titanium alloy metacarpal screw, the CoCr metacarpal head, the CFR-PEEK lined radial cup and the titanium alloy radius screw. Scale in mm.

At revision, only damaged or loose screws were removed; however, the articular components were revised in all cases. All articular components had a nominal diameter of 15 mm. Figure 1 shows the Motec 1 explant, to indicate the dimensions of the CoCr metacarpal head, the (black) CFR-PEEK lined radial cup and the metacarpal and radial titanium alloy screws. In terms of the length of the metacarpal head necks, five were long and two were medium. In addition, one Mo-CFR PEEK Motec was implanted as a revision of a MoM Motec for a periprosthetic metacarpal fracture.

Revision of implants was performed 0.5–3.8 years after implantation (mean 2.0) and was performed for a variety of reasons, as outlined in Table 1. Of the seven explants, two were revisions of primary replacements performed by one of the clinical authors (DB), one was a second revision (DB) and four were revisions of primary surgery performed elsewhere.

**Figure 2.**
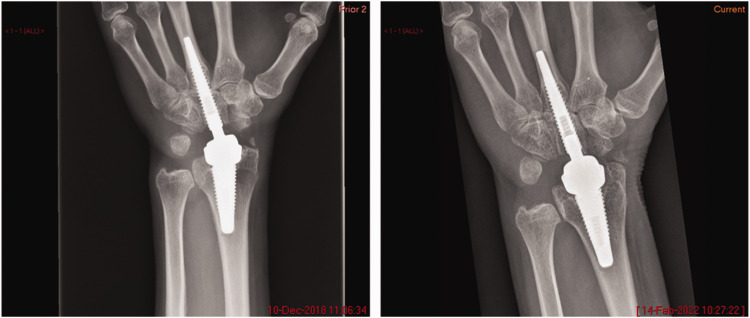
Radiographs of a well-fixed metacarpal stem of Motec 4 (left) and a later radiograph showing osteolysis and implant subsidence (right).

Two of the Motec implants, 4 and 6, were explanted for osteolysis after previously well-fixed implants. Radiographs of Motec implant 4 showing initial well-fixed implant and osteolysis are shown in Figure 2. At the time of explantation, there was discoloured synovial tissue which, upon histological examination, demonstrated aggregates of histocytes containing particles of polymer. Motec implants 1–4 and 7 were revised to further Motec TWA; Motec implants 5 and 6 were revised to fusion. No short-term issues were reported after the revision surgeries, and good short-term results were achieved.

**Figure 3.**
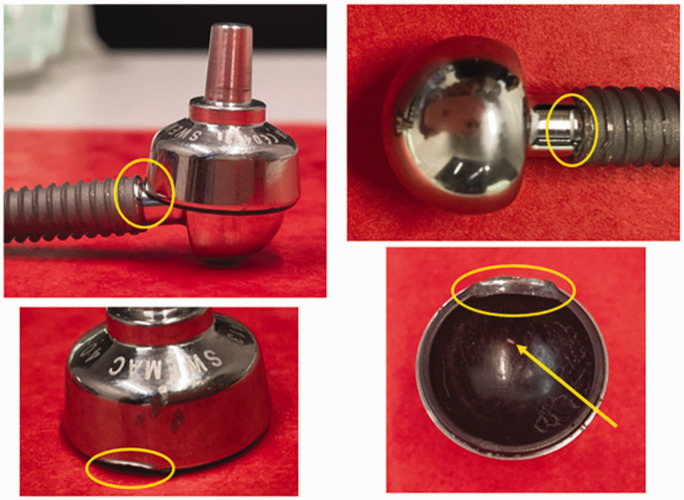
A composite image of Motec 4 explant showing the impingement (indicated by yellow circles) between the circumference of the radial cup and the stem of the metacarpal head. Note the circumferential damage (length approximately 8 mm) at the top of the bottom right image. A relatively large metallic wear particle appears to be embedded in the CFR-PEEK (bottom right image, identified by yellow arrow).

**Figure 4.**
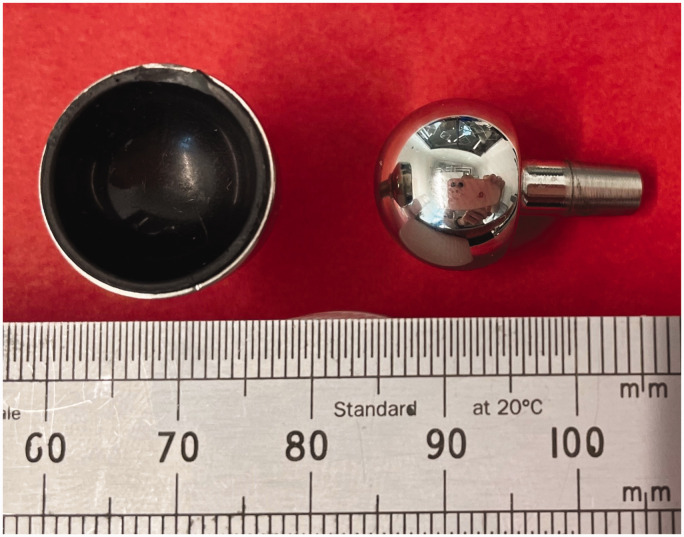
The metacarpal head (right) and CFR-PEEK radial cup of Motec 6 explant. Note the rim damage at the top of the CFR-PEEK cup.

From a visual analysis, there were no signs of the damage to any of the three metacarpal screws as seen in impingement-related osteolysis, as reported by Julian et al. (2024). This may have been expected given that there were no short neck samples, but it is reassuring that impingement-related osteolysis has not been seen with medium and long neck samples. There was, however, some damage to the rims of two CFR-PEEK cups (Motec explants 4 and 6). One of these (Motec explant 4) is shown in composite Figure 3. Figure 4 shows a view of the cup and head of Motec explant 6; again, circumferential damage can be seen on the cup.

Regarding the surface topographical analysis, the average roughness measured from the articulating surfaces of the components of the unworn ‘Motec 0’ and the explants is provided in Table 2. Measurements of the articulating surfaces showed a change in skewness from positive to negative. For the explanted CoCr metacarpal heads, five of seven explanted heads showed a statistically significant decrease in roughness compared with a new sample. For the explanted CFR-PEEK radial cups, three of seven explanted cups showed a statistically significant decrease in roughness compared with a new sample.

**Table 2.** Surface roughness (Sa) and skewness (Ssk) of the heads and cups of the unused and explanted Motecs.

Explant	Mean CoCr head roughness (µm)	Mean CFR PEEK cup roughness (µm)	Mean head skewness	Mean cup skewness
Motec 0 (unused)	0.030 (0.008)	2.555 (0.635)	0.588 (0.785)	1.092 (0.756)
Motec 1	0.017 (0.007)***p* < 0.05**	3.110 (0.362)***p* < 0.05**	−0.092 (0.109)	−2.263 (1.185)
Motec 2	0.028 (0.018)*p* = 0.63	2.211 (0.330)*p* = 0.26	−0.502 (0.207)	−1.538 (1.085)
Motec 3	0.016 (0.005)***p* < 0.05**	2.415 (0.337)*p* = 0.56	−0.055 (0.274)	−1.424 (0.541)
Motec 4	0.024 (0.009)*p* = 0.29	0.569 (0.331)***p* < 0.05**	−0.271 (0.124)	−2.145 (1.194)
Motec 5	0.008 (0.004)***p* < 0.05**	1.723 (0.281)***p* < 0.05**	−0.769 (0.426)	−1.923 (0.971)
Motec 6	0.019 (0.010)***p* < 0.05**	2.205 (0.203)*p* = 0.18	−0.122 (0.109)	−1.719 (0.442)
Motec 7	0.020 (0.004)***p* < 0.05**	0.990 (0.339)***p* < 0.05**	−0.100 (0.098)	−1.936 (0.378)

Data are expressed as mean (SD). The *p*-values calculated from the statistical comparison of the average roughness values of the unused head and cup with the explanted heads and cups. The values in bold indicate statistically significant results.

CFR-PEEK: carbon fibre reinforced poly ether ether ketone; CoCr: cobalt chromium; SD: standard deviation.

## Discussion

From our analysis of explanted Mo-CFR-PEEK Motec TWAs, we found no major damage on the articulating surfaces. Some rim damage was seen in two of seven samples, and this appears to be associated with osteolysis.

Despite the reported good results with MoM Motec TWA (Giwa et al., 2018; Reigstad et al., 2017; Reigstad et al., 2012), hand surgeons performing wrist arthroplasty may have opted for the Mo-CFR-PEEK Motec TWA, due to historical wear-related failures in some MoM hips (Langton et al., 2010). Aside from the radial cup rim damage seen in two cases, our analysis did not demonstrate any significant articular surface damage; instead, most of the articulating surfaces of the explanted components showed a decrease in roughness as well as a change to a negative skewness, compared with new samples, suggesting self-polishing.

From this cohort, only three of the primary surgeries were performed by the clinical authors, while the majority had had their surgery performed in other centres and were referred in for revision. We have no information of the number of primary procedures performed in those other centres and cannot therefore calculate the early revision rate, which has been reported elsewhere as 8.6% at 5.8 years (Redfern et al., 2024).

Analysis of Motec explant 4 (Figure 3) showed rim damage due to impingement against the proximal edge of the metacarpal screw, as previously described (Karjalainen et al., 2018) and which was discussed in detail (Julian et al., 2024). In Motec explant 4, the medium neck implant predated the changes in neck lengths introduced in 2020 to address this issue, which meant that the cup rim and metacarpal screw cannot now impinge with any combination of cup types and screw sizes. In Motec explant 6, the medium neck was one of the newer designs, so the CFR-PEEK impinged against the side of the neck and has resulted in less damage. The company are aware of these issues and have designed a new version of the CFR-PEEK cup, where the CFR-PEEK liner sits below the level of the CoCr rim and so any impingement will be between the CoCr rim and the CoCr neck, which has been shown previously to cause less damage and debris (Kandemir et al., 2024). The release of this new cup is subject to Medical Device Regulations approvals.

Surface roughness (Sa) measurements serve as an indication of in vivo changes, while skewness (Ssk) measurements provided information on the tendency of the surfaces to have peaks or valleys. Unworn surfaces predominantly have positively skewed surfaces (peaks are dominant), whereas worn surfaces tend to have negatively skewed surfaces (valleys are dominant) (Scholes et al., 2013). All the articulating surfaces of the explants were negatively skewed, indicating that the initial peaks of the manufactured surfaces had been removed. Compared to the unused head, five of the explanted heads became smoother in vivo and compared to the unused cups, three cups became smoother in vivo (Table 2). The surfaces that became smoother suggested self-polishing, which tend to produce less wear (Sieber et al., 1999). All of the metal head roughness values were less than 50 nm Sa, which is likely the upper value of manufacturing tolerances (International Standards Organisation, 2009). There is no equivalent standard for the roughness of CFR-PEEK articulating surfaces.

The biomaterial CFR-PEEK may be familiar to hand surgeons as a material used in bone anchors, and instrumentation and implants for distal radial fractures (Tarallo et al., 2020). However, its use as a bearing material in joint replacements is less established. Previously, Arevalo et al. (2023) looked at disparate findings of multiple in vitro tribological studies concerning CFR-PEEK and noted a need for more retrieval studies. Hopefully, our study has contributed to the literature in this regard. Concerning the articulation of CoCr against CFR-PEEK, Kandemir et al. (2019) found low wear of the CFR-PEEK counterface, but that this was negated by wear of the CoCr counterface. The authors concluded that CFR-PEEK should not be used in an articulation with orthopaedic metals (Kandemir et al., 2019). In another in vitro study concerned with the bushes of hinged knee joint replacements made of CFR-PEEK, the authors concluded that the use of CFR-PEEK against CoCr should be avoided (Schroeder et al., 2020). To add to this pessimism, the authors of a clinical study concerning CFR-PEEK in hip joint replacements did not recommend the use of CFR-PEEK acetabular liners (Heijnens et al., 2021). These negative findings were not seen in our short-term study. Our short-term findings are in agreement with another recent paper concerned with rotating hinge total knee prostheses, which found no adverse events with CFR-PEEK (Vertesich et al., 2022). While the wrist is a different application to the hip and the knee, in that the joint loading is far lower, there remains a need for a full evaluation of novel biomaterials used clinically as bearings such as CFR-PEEK.

A limitation of this study was that the volumetric wear of the prostheses was not measured as this is a major challenge for such explants and has not previously been performed on such small (15 mm diameter) components. However, the clinical data suggest that wear is not a problem over the timescales considered, in that we did not find any metallosis, adverse reaction to particulate debris or pseudotumours in patients where impingement was not seen. Moreover, the skewness data and most of the roughness data, on all explants, further imply that surfaces self-polished. Therefore, we are of the view that the roughness and clinical data serve as surrogate indicators of the lack of substantial wear of Mo-CFR PEEK Motec TWAs, at least over the short-term follow-up duration reported here.

In conclusion, our short-term explant study did not find any major damage on the articulating surfaces of any of the explanted prostheses. For many components, self-polishing occurred in vivo. Two of the seven explanted radial cups showed rim damage, presumably due to impingement with the metacarpal component. Explant analysis offers important insights into the performance of contemporary TWAs and adds to the overall understanding of novel biomaterials and designs of artificial joints. In this study, we have not identified any concerning issues with bearing surfaces of the Mo-CFR-PEEK Motec implant.
